# A case of relieving thymoma after conservative management of anti‐gamma‐aminobutyric acid receptor type A encephalitis

**DOI:** 10.1111/1759-7714.15402

**Published:** 2024-07-10

**Authors:** Yanmei Li, Ning Xie

**Affiliations:** ^1^ Department of Rheumatology Qilu Hospital, Cheeloo College of Medicine, Shandong University Jinan China; ^2^ Department of Rheumatology Yantaishan Hospital Yantai China; ^3^ Department of Chest Surgery Yantaishan Hospital Yantai China

**Keywords:** encephalitis, conservative treatment, gamma‐aminobutyric acid receptor type A, thymoma

## Abstract

Anti‐gamma‐aminobutyric acid receptor type A (GABAA) encephalitis is a relatively rare autoimmune encephalitis, and often associated with thymoma. Here, a 44‐year‐old female was diagnosed as having a thymoma with autoimmune encephalitis. At 4‐month follow‐up she was without recurrence of symptoms after treatment with methylprednisolone pulse therapy and immunotherapy. This case report provides a reference for the identification of this type of paraneoplastic encephalitis and for a therapeutic schedule. It also highlights that conservative treatment may be effective for patients with a tumor and GABAA encephalitis.

## Graphical abstract

Chest enhanced computed tomography (CT) on admission. (a) Axial plane. (b) Coronal plane. (c) Sagittal plane. 127 × 42 mm (300 × 300 dpi). Repeated chest enhanced CT 4 months later. (d) Axial plane. (e) Coronal plane. (f) Sagittal plane. 127 mm × 42 mm (300 × 300 dpi).

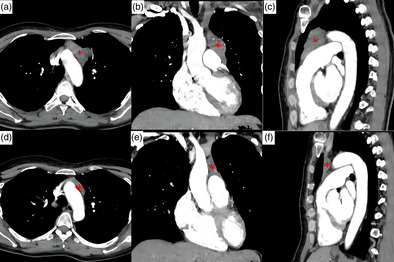

## INTRODUCTION

Paraneoplastic encephalitis includes different syndromes that may be triggered by occult tumors.^1^ Anti‐gamma‐aminobutyric acid receptor type A (GABAA) encephalitis is a relatively rare autoimmune encephalitis, which often presents with seizures and almost one‐third of patients have an associated tumor especially thymoma.^2^, ^3^ Here, we present a patient with thymoma associated with GABAA encephalitis who received methylprednisolone pulse therapy and immunotherapy. This case report provides a reference for the identification of this type of paraneoplastic encephalitis and for a therapeutic schedule.

## CASE REPORT

**FIGURE 1:**
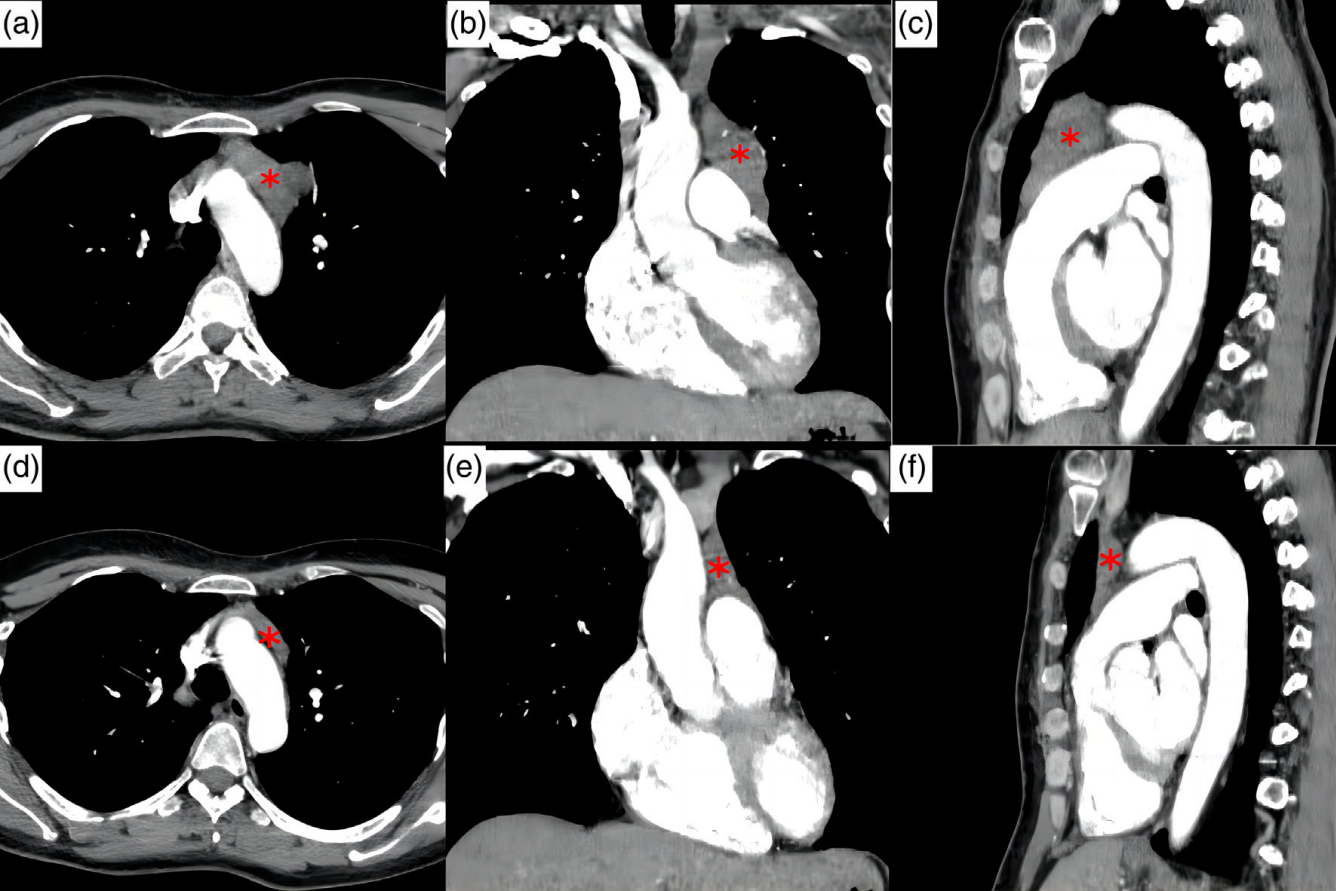
Chest enhanced computed tomography (CT) on admission. (a) Axial plane. (b) Coronal plane. (c) Sagittal plane. 127 mm × 42 mm (300 × 300 dpi). Repeat chest enhanced CT 4 months later. (d) Axial plane. (e) Coronal plane. (f) Sagittal plane. 127 mm × 42 mm (300 × 300 dpi).

A 44‐year‐old female was admitted to the Department of Rheumatology with an oral ulcer, alopecia and facial erythema for more than 1 month. Upon admission, her temperature was 36.5°C, heart rate was 82 beats/min, respiratory rate was 20 breaths/min, and blood pressure was 125/78 mmHg. The patient was antinuclear antibody (ANA) (ANA1:320) positive, anti‐ds‐DNA antibody negative, complement C3 was 0.55 g/L, complement C4 was within the normal range, except for systemic lupus erythematosus, sudden limb convulsions, loss of consciousness, and eyes staring to the right during the examination. The patient's symptoms lasted 3 min. Head computed tomography (CT) showed no abnormalities. However, chest CT revealed an anterior mediastinal mass. Subsequent chest enhanced CT (Figure 1a–c) showed the mass with moderate inhomogeneous enhancement (asterisk), measuring about 4.5 cm × 3.0 cm × 5.9 cm. Laboratory evaluation revealed the following: neuron‐specific enolase, 22.05 ng/mL (reference range, 0.0–16.3 ng/mL); cytokeratin 19 fragment (CYFRA 21‐1), 3.43 ng/mL (reference range, 0.0–3.3 ng/mL). Other tumor markers were normal, including alpha fetoprotein, carcinoembryonic antigen and cancer antigen 199. Head magnetic resonance imaging (MRI) examination revealed no obvious lesion. The patient suffered seizures twice after admission, and a lumbar puncture was then performed. Further cerebrospinal fluid tests showed that the GABAA receptor antibody IgG was positive. Combined with the anterior mediastinal mass and positive GABAA receptor antibody, the patient was diagnosed as having a thymoma with autoimmune encephalitis. After methylprednisolone 1000 mg once daily for 3 days, changed to 500 mg once daily for 3 days, 240 mg/day for 3 days, 120 mg/day for 3 days, then changed to prednisone 60 mg once daily and regularly reduced to 10 mg once a day, mycophenolate mofetil capsules 500 mg twice daily was administered along with glucocorticoids. At 4‐month follow‐up, she was without recurrence of symptoms. Repeat chest enhanced CT (Figure 1d–f) showed the mass had significantly reduced in size (asterisk), measuring about 3.3 cm × 1.2 cm × 3.5 cm.

## DISCUSSION

GABAA encephalitis has been recognized as a distinct antibody‐mediated autoimmune encephalitis, which may be associated with malignancy detected by the time of diagnosis, especially thymoma.^2^ Serum and cerebrospinal fluid cell‐based assay testing are often used for detecting anti‐GABAA receptor antibody.^3^ For GABAA encephalitis, the majority of patients had partial to complete recovery in response to antiepileptic treatment and immunotherapy.^3^ Treatment options include first‐line immunotherapy (intravenous methylprednisolone, intravenous gammaglobulin, and/or plasmapheresis), second‐line immunotherapy (including rituximab, azathioprine, mycophenolate mofetil, cyclophosphamide, and/or tacrolimus), and antiepileptic treatment. Partial to complete recovery is seen in the majority of patients who receive either first or both first‐ and second‐line immunotherapy. Delayed treatment may lead to severe and permanent neurological deficits.^4^, ^5^ Recognition of this type of paraneoplastic encephalitis is important. There were some limitations to this study. Based on the patient's initial condition on admission, selective operation resection of the mediastinal mass was planned. However, the mass subsequently reduced in size. In the case reported here there was a lack of pathology and the specific pathological type of the mediastinal mass was unknown. This study highlights that when thymoma is associated with GABAA encephalitis, conservative treatment may also be effective to the tumor.

## AUTHOR CONTRIBUTIONS

Yanmei Li and Ning Xie: Conceived the study. Ning Xie: Collected the data. Yanmei Li: Wrote the manuscript. All authors read and approved the final version of the manuscript.

## CONFLICT OF INTEREST STATEMENT

The authors have no actual or potential conflicts of interest to declare.

## Data Availability

All other data are available from the corresponding author (or other sources, as applicable) on reasonable request.
